# *ATP1A1* Mutant in Aldosterone-Producing Adenoma Leads to Cell Proliferation

**DOI:** 10.3390/ijms222010981

**Published:** 2021-10-12

**Authors:** Kazuhiro Kobuke, Kenji Oki, Celso E. Gomez-Sanchez, Elise P. Gomez-Sanchez, Kiyotaka Itcho, Haruya Ohno, Gaku Nagano, Yoko Yoshii, Ryuta Baba, Takaya Kodama, Koji Arihiro, Noboru Hattori, Masayasu Yoneda

**Affiliations:** 1 Department of Molecular and Internal Medicine, Graduate School of Biomedical and Health Sciences, Hiroshima University, Hiroshima 734-8551, Japan; kazu-kobuke@hiroshima-u.ac.jp (K.K.); itcho@hiroshima-u.ac.jp (K.I.); haruya-ohno@hiroshima-u.ac.jp (H.O.); gnagano@hiroshima-u.ac.jp (G.N.); yoko.yoshii56@gmail.com (Y.Y.); rtbaba45@gmail.com (R.B.); kktkykk.88@gmail.com (T.K.); nhattori@hiroshima-u.ac.jp (N.H.); masayone17@hiroshima-u.ac.jp (M.Y.); 2 Division of Endocrinology, G.V. (Sonny) Montgomery VA Medical Center and University of Mississippi Medical Center, Jackson, MS 39216, USA; cgomez-sanchez@umc.edu; 3 Department of Pharmacology & Toxicology, University of Mississippi Medical Center, Jackson, MS 39216, USA; egomez-sanchez@umc.edu; 4 Department of Anatomical Pathology, Hiroshima University Hospital, Hiroshima 734-8551, Japan; arihiro@hiroshima-u.ac.jp

**Keywords:** primary aldosteronism, aldosterone-producing adenoma, Na/K-ATPase, cell proliferation, cardiotonic steroid

## Abstract

The molecular mechanisms by which *ATP1A1* mutation-mediated cell proliferation or tumorigenesis in aldosterone-producing adenomas (APAs) have not been elucidated. First, we investigated whether the APA-associated *ATP1A1* L104R mutation stimulated cell proliferation. Second, we aimed to clarify the molecular mechanisms by which the *ATP1A1* mutation-mediated cell proliferated. We performed transcriptome analysis in APAs with *ATP1A1* mutation. *ATP1A1* L104R mutation were modulated in human adrenocortical carcinoma (HAC15) cells (ATP1A1-mutant cells), and we evaluated cell proliferation and molecular signaling events. Transcriptome and immunohistochemical analysis showed that Na/K-ATPase (NKA) expressions in *ATP1A1* mutated APA were more abundant than those in non-functioning adrenocortical adenoma or *KCNJ5* mutated APAs. The significant increase of number of cells, amount of DNA and S-phase population were shown in ATP1A1-mutant cells. Fluo-4 in ATP1A1-mutant cells were significantly increased. Low concentration of ouabain stimulated cell proliferation in ATP1A1-mutant cells. ATP1A1-mutant cells induced Src phosphorylation, and low concentration of ouabain supplementation showed further Src phosphorylation. We demonstrated that NKAs were highly expressed in *ATP1A1* mutant APA, and the mutant stimulated cell proliferation and Src phosphorylation in ATP1A1-mutant cells. NKA stimulations would be a risk factor for the progression and development to an *ATP1A1* mutant APA.

## 1. Introduction

Primary aldosteronism (PA) is the most common form of secondary hypertension, with a prevalence of eight to ten percent among hypertensive patients. It is associated with a significant increase in cardiovascular morbidity and mortality as compared to that in essential hypertension with similar levels and duration of elevated blood pressure [1,2,3]. PA is mainly classified with aldosterone-producing adenoma (APA) and idiopathic hyperaldosteronism [4]. Somatic mutations of *KCNJ5*, *ATP1A1*, *ATP2B3*, *CACNA1D*, *CACNA1H*, *CLCN2*, or *CTNNB1* genes that drive autonomous aldosterone production have been documented in APAs [5,6,7,8,9,10,11,12,13]. Recently, Gong, et al., suggested that metabolic reprograming involved with tumorigenesis in *KCNJ5* mutant APA [14]. However, the molecular mechanisms of genesis or growth of APAs mediated by *ATP1A1* mutations have not been elucidated.

Aldosterone-producing cell clusters (APCCs), also called Aldosterone-producing micronodules (APM), are foci of cells with zona glomerulosa (ZG) morphology, and strong and uniform immunoreactivity for aldosterone synthase (CYP11B2) [15,16]. *ATP1A1* mutations found in APA have also been detected in APCCs of normal individuals and in APCC-to-APA transitional lesions (AATLs), which have similar characteristics to APCC with respect to CYP11B2 immunoreactivity and penetrate into the zona fasciculata (ZF) [17,18]. Recent evaluation of the accumulated mutations and histopathology of the adrenal glomerulosa carried a potential that APM are the origin of APAs [19,20]. On the other hand, two-hit model for APA formation was also described in cases with APC variant or with partial and well-localized KCNJ5 staining in APA [21,22,23]. Therefore, the mechanism of APA development is still uncertain and may differ depending on the underlying mutation(s).

Na/K-ATPase (NKA) is a transmembrane heterodimer composed of a catalytic α subunit and a glycosylated β subunit, which exchange intracellular Na^+^ for extracellular K^+^ using ATP as its source of energy. The catalytic α subunit of NKA appears in four different forms, and α1 subunit is coded by the *ATP1A1* gene. The β subunit controls α/β heterodimer assembly and insertion into the plasma membrane [24]. The roles of NKA by exchange of Na^+^ and K^+^ are the responsible for the osmoregulation as well as the transmission of nerve impulses, and cardiotonic steroids with pharmacologic concentration, micromolar range, inhibits the pump function [25]. The mutations of *ATP1A1*, G99R, L104R, V332G, and EETA963S, are located in transmembrane domain of NKA [5,6,26,27]. Those caused the alteration of K^+^ binding and loss of pump activity, and resulted in membrane depolarization and voltage-gated Ca^2+^ channel activation. In addition to the pump function, the NKA also works as a receptor, and NKA consists of receptor complex coupled with proto-oncogene non-receptor tyrosine-protein kinase (Src) for signal transduction. The signal transduction is triggered by low nanomolar concentration of cardiotonic steroids, which does not influence the pump function [25,28]. NKA-associated Src phosphorylation activates intracellular signaling molecules including epidermal growth factor receptor (EGFR), Inositol 1,4,5-trisphosphate receptor (IP3R), phospholipase C-γ (PLC-γ), and phosphatidylinositol 3-kinase (PI3K), which promote increased cell volume, cell proliferation, and tumor progression [29,30,31,32]. 

Known signaling properties of NKA together with the mutational analysis and the molecular evidence mentioned above led us to hypothesize that the somatic mutations of the *ATP1A1* gene induce tumorigenesis and stimulate tumor growth of APA in addition to increasing autonomous aldosterone synthesis. In the initial part of our study, we determined whether an *ATP1A1* mutation induced cell proliferation in vitro. This was followed by exploring key molecules that promote cell proliferation using APA samples to elucidate the molecular mechanism of APA tumorigenesis. Our data revealed that other subfamilies of ATPase were upregulated in APAs with *ATP1A1* mutations. The signal transduction functions of one or more NKA that are upregulated in APAs might be responsible for tumorigenesis or tumor growth. We thus hypothesized that increased total NKA-associated signal transduction might potentiate cell proliferation in *ATP1A1* mutant APA. The aim of our study was to delineate the mechanism by which NKA signal transduction induces proliferation in *ATP1A1*-mutatant adrenal cells. The *ATP1A1* L104R mutation was selected as the prototype because it is reasonably common among the *ATP1A1* mutations documented in APAs and functions in a similar manner as other *ATP1A1* mutations.

## 2. Results

### 2.1. Transcriptome Analysis in APA with ATP1A1 Mutation

**Figure 1:**
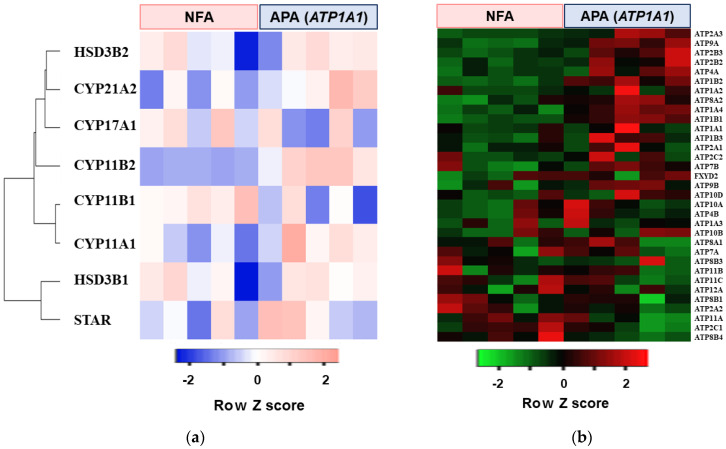
Heat map of concerns genes in five aldosterone–producing adenomas (APA) with an *ATP1A1* mutation and five non–functioning adrenocortical adenomas (NFA). (**a**) Heat map of steroidogenic enzymes genes. (**b**) Heat map of genes related to P type ATPase function.

Microarray analysis was performed using five APAs with *ATP1A1* mutations and five non–functioning adrenocortical adenomas (NFAs). *ATP1A1* mutant APAs had higher levels of steroidogenic enzymes including *CYP11B2* expression relative to NFAs (Figure 1a). Pathway analysis with GSEA was performed by R software. The top five pathways up-regulated in *ATP1A1*-mutated APAs are shown in Table 1. The ion transport pathway by P type ATPase was given specific emphasis, as high expression of NKA may stimulate the cell proliferation or APA growth. The comparison of the expression rates of the genes for all the subunits for human P type ATPases between *ATP1A1*-mutated APA and NFA are shown in Figure 1b and Table 2. Several genes including *ATP1A2*, *ATP1A4*, *ATP1B1,* and *ATP1B2*, which encode for α- and β-subunit of NKA, were highly expressed in *ATP1A1* mutant APAs. 

### 2.2. Immunohistochemical Analysis of NKA in APAs and NFAs

**Figure 2:**
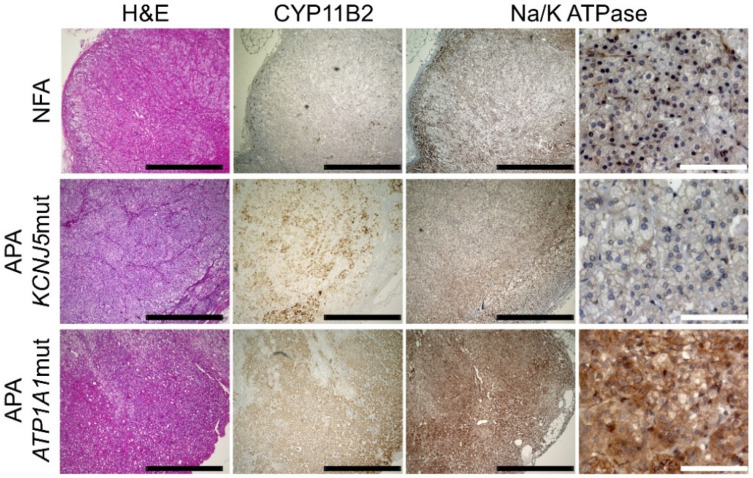
Immunohistochemical detection of sodium/potassium-transporting ATPase (NKA) in aldosterone-producing adenomas (APA) with an *ATP1A1* or *KCNJ5* mutation and a non-functioning adrenocortical adenoma (NFA). Black and white scale bars indicate 1 mm and 100 μm, respectively.

Since genes coding for NKA subunits were highly expressed in APAs with an *ATP1A1* mutation, we performed immunohistochemical analysis of NKA in *ATP1A1* and *KCNJ5*-mutated APA and NFA. To know the expression of all the NKA in the adenoma, a polyclonal antibody for total NKA was applied in this experiment. NKA positive cells were more abundant in *ATP1A1* mutant APAs as compared to those in *KCNJ5* mutant APAs and NFAs (Figure 2). 

### 2.3. Effects of ATP1A1 Mutation on NKA Expression in Human Adrenocortical Carcinoma (HAC15) Cells

**Figure 3:**
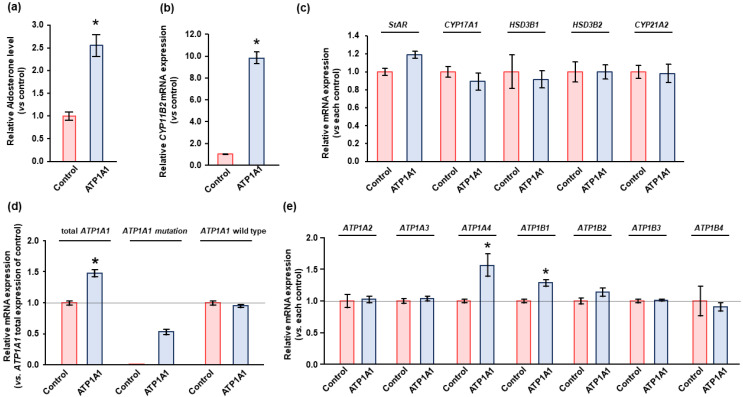
Effect of *ATP1A1* L104R mutation on aldosterone production and genes related with Na/K-ATPase (NKA) in HAC15 cells. (**a**,**b**) After transduction of *ATP1A1* mutant (*n* = 3) or control (*n* = 3) in HAC15 cells and attainment of confluency on 24-well plates, cells were serum deprived in DMEM/F12 containing 0.1% serum for 24 h. Cells were incubated with fresh media with 0.1% serum for 24 h. Aldosterone levels of the supernatant and *CYP11B2* expression in the cells were measured. *, *p* < 0.05 vs. control cells. (**c–e**) After transduction of *ATP1A1* mutation (*n* = 3) or control (*n* = 3) and incubation with DMEM-F12 containing 0.1% cosmic calf serum on 24-well plates for 24 h. Total RNA was extracted, and RNA sequencing analysis was performed. mRNA levels of steroidogenic enzymes were determined as relative expression of each control. Total *ATP1A1*, *ATP1A1* mutation, and *ATP1A1* wild type expression levels were determined as relative expression of total *ATP1A1* in control cells. *, *p* < 0.05 vs. total *ATP1A1* expression of control cells. Gene expression levels of NAK subfamily were also measured by RNA sequencing analysis. *, *p* < 0.05 vs. each control cells.

There was a significant increase in basal aldosterone production by 2.5-fold and in *CYP11B2* transcripts by 9.8-fold in HAC15 cells stably expressing the *ATP1A1* mutant relative to control cells (Figure 3a,b). Transduction with the wild type *ATP1A1* lentivirus did not alter aldosterone biosynthesis or *CYP11B2* transcripts compared to transduction with the empty lentivirus (data not shown). Further, the gene expression levels of *StAR* and the steroidogenic enzymes *CYP17A1*, *HSD3B1*, *HSD3B2*, and *CYP21A2* were not altered by the *ATP1A1* mutant in HAC15 cells (Figure 3c). To evaluate the effect of increased NKA expression due to *ATP1A1* mutation on cell proliferation, we introduced empty vector as control in HAC15 cells. Gene expression analysis demonstrated that HAC15 cells with *ATP1A1* mutation showed 1.36-fold increase of total *ATP1A1* expression (*p* < 0.05), and native *ATP1A1* expression did not change after the introduction of *ATP1A1* mutant (Figure 3d). The *ATP1A1* mutation significantly increased *ATP1A1* and *ATP1B1* expression were increased in HAC15 cells (Figure 3e).

### 2.4. Effects of ATP1A1 Mutation on Cell Proliferation in HAC15 Cells

**Figure 4:**
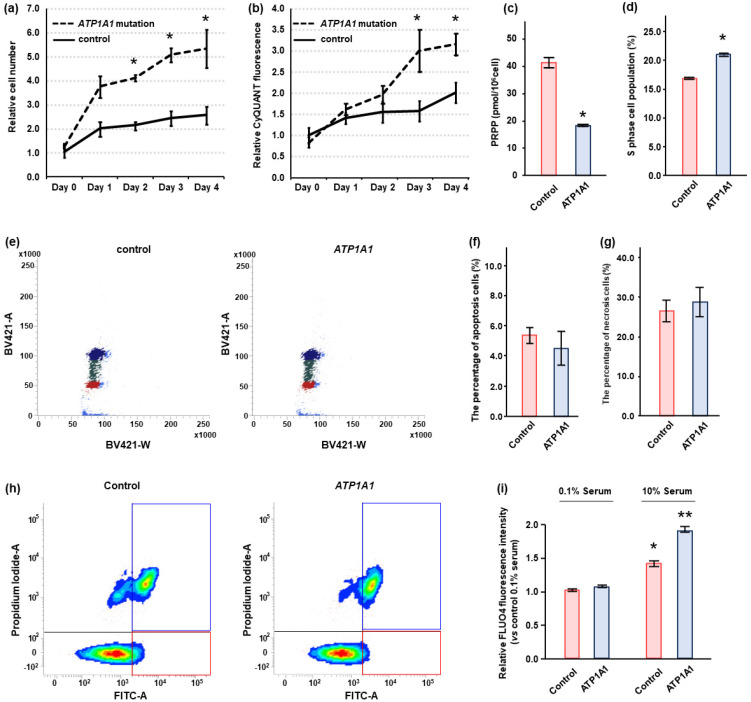
Effect of *ATP1A1* L104R mutation on cell proliferation in HAC15 cells. (**a**,**b**) HAC15 cells with 40–50% confluency on 96-well plates were transduced with a lentivirus *ATP1A1* mutant (*n* = 6) or control (*n* = 6) vectors. The cells were cultured in DMEM/F12 containing 10% serum. The effects of the *ATP1A1* mutant on cell proliferation and DNA amounts were evaluated by counting cell number using TC20 Cell Counter at the indicated times and CyQUANT Direct Cell Proliferation Assay Kit, respectively. *, *p* < 0.05 vs. each type of control cells. (**c**) After transduction of *ATP1A1* mutant (*n* = 3) or control (*n* = 3) in HAC15 cells and attainment of confluency. Cells were incubated in DMEM/F12 containing 10% serum on 6-well plates for 24 h, and Phosphoribosyl Diphosphate (PRPP) levels in cells were measured by capillary electrophoresis time-of-flight mass spectrometry. (**d**,**e**) After transduction of *ATP1A1* mutant (*n* = 3) or control (*n* = 3) in HAC15 cells and attainment of confluency. Incubation with DMEM-F12 containing 10% cosmic calf serum on 6-well plates for 48 h, cells were treated with 10 μM of cycloheximide for 24 h. The cells with supplemental solutions of Cell Cycle Assay Solution Blue were injected into the flow cytometer instrument, and the cells of G0/G1, S, or G2/M phase were shown in the figure with red, green, or blue, respectively. The number of cells of S phase were compared. *, *p* < 0.05 vs. control cells. (**f**–**h**) After transduction of *ATP1A1* mutant (*n* = 3) or control (*n* = 3) and incubation with DMEM-F12 containing 10% cosmic calf serum on 6-well plates for 72 h, cells were harvested. Following Promokine Apoptotic/Necrotic Cells Detection kit (PromoCell, Heidelberg, Germany), the cells (2.5 × 10^5^) were incubated with Annexixn and Ethidium Homodimer for 15 m. The samples containing cells were injected into the flow cytometer instrument (BD FACSAriaIIu, BD Biosciences, Franklin Lakes, NJ, USA). The results of control and *ATP1A1* mutation are depicted in A and B, respectively. The cells in red and blue squares were defined as apoptotic and necrotic, respectively. The apoptotic and necrotic cell number per total cell number were compared between control and *ATP1A1* mutation cells. (**i**) After transduction of *ATP1A1* mutant (*n* = 4) or control (*n* = 4) vectors in HAC15 cells and incubation with DMEM/F12 containing 0.1% serum for 24 h, cells were incubated with fresh media with or without 10% serum with 3 μM of Fluo4-AM on 96-well plates for 10 m. *, *p* < 0.05 vs. control cells with 0.1% serum. **, *p* < 0.05 vs. other three type of cells.

We counted cell number and calculated DNA quantity in control and HAC15 cells with the *ATP1A1* L104R mutant. When grown in regular media, the cell number and DNA quantity in cells expressing the *ATP1A1* mutation became significantly greater than that in control cells within two to three days after gene transduction (Figure 4a,b). Phosphoribosyl Diphosphate (PRPP), which was consumed by the increase of purine and pyrimidine nucleotide biosynthesis [33], was measured in control and *ATP1A1* mutant cells. PRPP level in *ATP1A1* mutant cells was lower than that in control cells (Figure 4c). The *ATP1A1* mutation significantly increased S-phase cell population by cell cycle analysis (Figure 4d,e). The *ATP1A1* mutation did not increase apoptotic or necrotic cells in HAC15 cells (Figure 4f–h). However, there was no differences in cell proliferation between *ATP1A1* mutated and control cells, when the amount of serum in the medium was reduced from 10% to 0.1% (data not shown). 

HAC15 cells with the *ATP1A1* mutation did not exhibit an increase in Fluo-4, an indicator of intracellular Ca^2+^ concentration, relative to control cells under 0.1% serum condition (Figure 4i). However, *ATP1A1* mutation led to a 1.3-fold increase in Fluo-4 levels as compared with control cells treated with 10% serum (*p* < 0.05, Figure 4i). These results indicated that serum might be essential for the increase of intracellular Ca^2+^ levels.

### 2.5. Effects of Ouabain in HAC15 Cells with ATP1A1 Mutation

**Figure 5:**
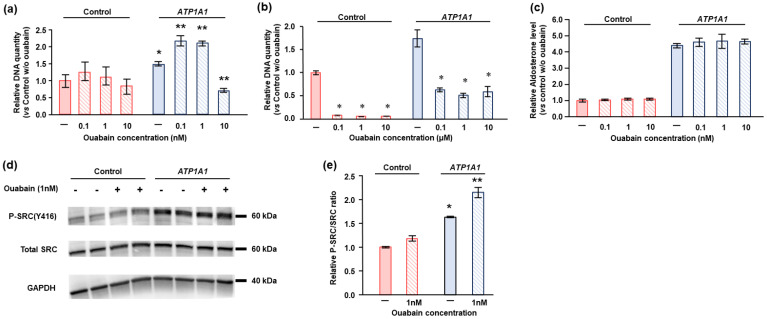
Ouabain enhanced cell proliferation of HAC15 cells with the *ATP1A1* L104R mutant, but did not increase aldosterone production. (**a**) After transduction of the *ATP1A1* mutant (*n* = 6) or control (*n* = 6) vectors in HAC15 cells and incubation with or without ouabain at the indicated concentration on 96-well plates for 3 days, the amount of DNA was measured by CyQUANT Direct Cell Proliferation Assay Kit. *, *p* < 0.05 vs. each type of control cells. (**b**) After transduction of *ATP1A1* mutant (*n* = 3) or control (*n* = 3) vectors in HAC15 cells and incubation with or without ouabain at the indicated concentration for 3 days, cells were serum deprived in DMEM/F12 containing 0.1% serum on 24-well plates for 24 h. Cells were incubated with fresh media with 0.1% serum for 24 h, and then aldosterone levels of the supernatant were measured. Aldosterone levels were normalized based on cellular protein levels. *, *p* < 0.05 vs. each control cells. (**c**) After transduction of *ATP1A1* mutation (*n* = 6) or control (*n* = 6) and incubation with or without ouabain at indicated concentration on 96-well plates for 3 days, the amounts of DNA were measured by CyQUANT Direct Cell Proliferation Assay Kit. *, *p* < 0.05 vs. each control cells. (**d**,**e**) After transduction of *ATP1A1* mutant (*n* = 3) or control (*n* = 3) vectors in HAC15 cells and incubation with DMEM/F12 containing 0.1% serum on 12-well plates for 24 h, cells were incubated with fresh media including 10% serum with or without 1 nM of ouabain for 5 m. *, *p* < 0.05 vs. control cells without ouabain. **, *p* < 0.05 vs. the other three type of cells.

Ouabain is a prototypical cardiotonic steroid commonly used for basic experiments with the NKA [28,34]. High concentrations of ouabain and other cardiotonic steroids inhibit NKA pump function, whereas lower concentrations stimulate cell proliferation via NKA-mediated signal transduction in cancer cells [28]. The effects of ouabain were studied in the control and *ATP1A1* L104R HAC15 cells grown in serum replete/deprived media. Low concentrations (0.1 nM to 1 nM) of ouabain significantly stimulate cell proliferation in HAC15 cells with the *ATP1A1* mutation, whereas it did not affect cell proliferation in control cells (Figure 5a). More than 10 nM of ouabain suppressed cell proliferation and decreased viable cells (Figure 5a,b). Ouabain did not stimulate aldosterone levels in *ATP1A1* mutant or control cells at any of the concentrations used (Figure 5c). 

To determine the mechanisms of NKA-mediated signal transduction on cell proliferation, we investigated the phosphorylation levels of Src, the major signaling molecule for NKA, by Western blotting. *ATP1A1* mutant cells had significantly higher basal Src phosphorylation levels than those of control cells, and treatment with 1 nM of ouabain further increased Src phosphorylation levels as compared to *ATP1A1* mutant cells without ouabain supplementation (Figure 5d,e).

## 3. Discussion

We demonstrated that NKAs were highly expressed in APAs with *ATP1A1* mutations than in NFA or APAs with *KCNJ5* mutations. Transduction of the *ATP1A1* L104R mutation responsible for autonomous production of aldosterone in some APAs stimulated cell proliferation in HAC15 cells under 10% serum concentrations and low concentrations of ouabain. Furthermore, low concentration of ouabain treatment resulted in an increased Src phosphorylation in HAC15 cells with *ATP1A1* mutation in the presence of adequate serum.

Clinical studies based on mutational and histopathological analyses suggested a progressive relationship between APM formation and APAs expressing *ATP1A1* mutations [17,18]. Our results showing the stimulation of HAC15 adrenal cell proliferation in presence of the *ATP1A1* L104R mutation, further supports this impression. The human adrenal cortex is histologically divided into three layers, the ZG, ZF, and zona reticularis. Aldosterone is produced by ZG cells, which consist of compact cells distinct from lipid droplet laden ZF cells; however, the morphology and steroidogenic capacity of cells within different APAs are variable. The *ATP1A1* mutation was originally found in the screening of APAs with compact cells [6], and further pathological study indicated that *ATP1A1*-mutated APA tend to have compact cells [20,35,36,37]. Our current results provide one of the mechanisms that a ZG cell with a mutation in the *ATP1A1* gene may proliferate and develop an APA. 

In contrast, the genesis of APAs with *KCNJ5* mutations is likely to be different from that with *ATP1A1* mutations. A *KCNJ5* mutation have been rarely found in APM [17,38]. Most APAs with *KCNJ5* mutations predominantly consist of clear cells, rather than the normally compact ZG cells [35,36,37,39]. Moreover, as we have previously reported, a *KCNJ5* mutation did not increase proliferation of HAC15 cells [40]. The absence of trophic effects of the *KCNJ5* mutations suggests that APA cells bearing *KCNJ5* mutations essentially require an additional factor or mutation to cause adenoma formation [23]. Taken together, the pathophysiology differs between APAs with *ATP1A1* and *KCNJ5* mutations. 

**Figure 6:**
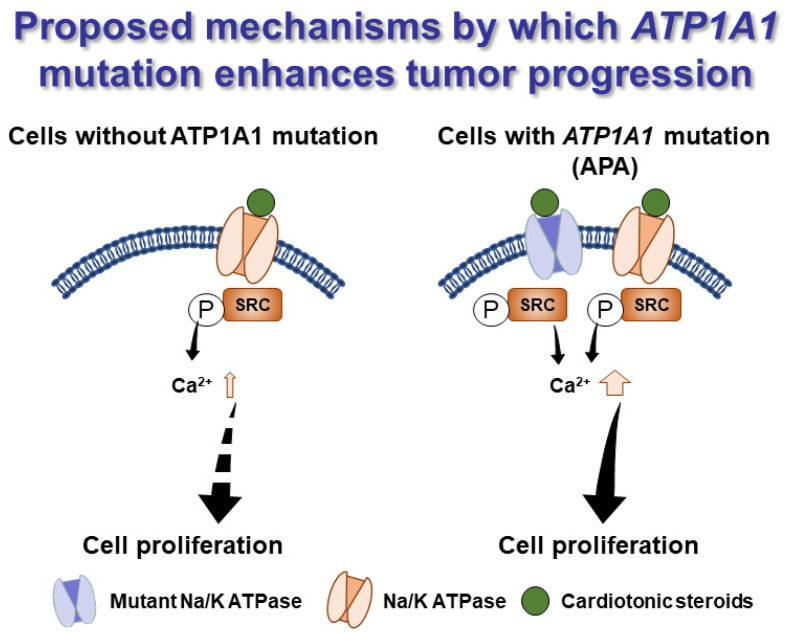
Hypothetical mechanism by which *ATP1A1* mutations promote tumor progression. Sodium/potassium-transporting ATPase (NKA) are more highly expressed in aldosterone-producing adenomas (APA) with an *ATP1A1* mutant to compensate for the loss of NKA pump function. This, subsequently, results in enhanced cell signaling function, including that of cell proliferation and intracellular Ca^2+^ concentration, upon stimulation of NKA with very low levels of a cardiotonic steroid.

We further showed that activation of Src through phosphorylation, one of the molecular mechanisms of NKA cell signaling, was enhanced in HAC15 cells with *ATP1A1* mutation and resulted in increased intracellular Ca^2+^ and cell proliferation, as summarized in Figure 6. APAs with *ATP1A1* mutations have abundant NKA levels, as other NKA subunit expression might be increased to compensate for decrease of NKA pump activity. α2 and α4 isoform of NKA, which were upregulated in HAC15 cells with *ATP1A1* mutation, have a higher or similar affinity for ouabain compared to α1 isoform of NKA [41,42]. We found that ouabain, a ligand of NKA, exerted a biphasic effect on *ATP1A1* mutant cell proliferation. Concentrations below or in the low nanomolar range increased proliferation of the mutated HAC15 cells via induction of a canonical NKA cell signaling mechanism. Higher doses of ouabain in the micromolar range inhibits the pump function of Na^+^ and K^+^ exchange. Our findings corroborate those of others. For instance, lower doses of ouabain were reported to have proliferative effects on adrenocortical tumor cells [43], and stimulate cell proliferation and progression in a number of cancer cell lines [44,45,46]. Collectively, these clinical and experimental data demonstrate that *ATP1A1* mutations in APA cells could induce cell growth via their cell signaling functions.

Cardiac glycosides including ouabain are naturally occurring compounds identified in various plant and animal species [47]. The existence of an endogenous ouabain or other cardenolide and its interaction with aldosterone were proposed over 40 years ago [48], and detection of cardiac glycosides in human plasma by High Performance Liquid Chromatography and Mass Spectrometry continues to be reported [49]. However, despite an enormous effort from many academic and commercial laboratories, the mechanism for the synthesis of ouabain or another cardenolide in a mammal remains unelucidated, nor have the intermediates or metabolites of such a compound been isolated [50,51]. Nevertheless, the concentrations of ouabain as low as 0.1 nM that induce NKA cell signaling pathways in other systems stimulated adrenal cell proliferation in our study as well [52,53], suggesting that cardiac steroids participate in the NKA-mediated cell proliferation too. If it is not endogenous, might the elusive ouabain-like compound sought by many be an environmental endocrine disruptor compound? The ZG of most normal adult human adrenal glands comprise many CYP11B2-immunoreactive APM surrounded by compact cells that do not express CYP11B2 [15,54], in addition to the fact that APM might be precursors for developing APAs with *ATP1A1* mutations. Consequently, measuring cardiac glycosides in plasma of the patients with APA may explain a potential role for a cardenolide regardless of its source. In addition, this knowledge may also facilitate the elucidation of preventive approaches for the development of APAs with an *ATP1A1* mutation. 

The effects of serum on cell proliferation were more prominent than those of ouabain. Therefore, mechanisms’ addition to NKA-mediated Src phosphorylation may be associated with *ATP1A1* mutation-induced cell proliferation. Since the aberrant or ectopic receptors such as MC2R, AVPR, and HTR4 are expressed in APA [55,56], the agonists including ACTH, arginine vasopressin, and somatostatin in serum might be associated with cell proliferation. It is reported that *ATP1A1* mutations, including the L104R, lead to decreased cytosolic pH in an adrenocortical cell line, resulting in autonomous aldosterone production [57]. Our pathway analysis showed that “Proximal Tubule Bicarbonate Reclamation” was upregulated in the *ATP1A1* mutant APA (Table 1). Intracellular acidification in *ATP1A1* mutant cells might potentiate gene transcription related with bicarbonate reclamation compensatory. Transmembrane protein 9 expression and Wnt/β-catenin signaling activation were associated with intracellular acidification induced colorectal cancer progression [58]. Therefore, intracellular acidification in *ATP1A1* mutant cells may also mediate APA development. We could not fully characterize the *ATP1A1* mutation mediated cell proliferation, and thus further basic investigations are needed. 

This study has some limitations. Sample size of this study is limited, because the frequency of *ATP1A1* mutation in APAs is small. Further studies are needed to confirm our results. Since this study was performed using one cancer-derived cell line, additional study using primary cell culture or cell lines from other species would be desired. Sufficient serum stimulated cell proliferation in HAC15 cells with *ATP1A1* mutation; however, we could not identify known cardiotonic steroids or other factors in the serum that influenced cell growth. Additionally, we could not test the effects of calcium signal inhibition or activation on cell proliferation in vitro. Further studies are required for the molecular mechanisms by which cardiotonic steroids and others may mediate APA development. 

## 4. Materials and Methods

### 4.1. Adrenal Tissues

The tissues of NFA (*n* = 5) and *ATP1A1* mutated APA (*n* = 5) were enrolled in this study. The clinical, pathological, and genetic characteristics were shown in a previous report [59], and no *CTNNB1* mutation was detected in NFA tissues. The presence of CYP11B2 by immunohistochemistry and quantitative polymerase chain reaction assays were confirmed in all APA tissues as previously reported [60,61]. This study was approved by the research ethics committee of Hiroshima University (Approval code; Hi-1 Hiroshima University, Hiroshima, Japan), and performed in accordance with the Declaration of Helsinki. All patients provided written informed consent. 

### 4.2. RNA Extraction and Quantitative Polymerase Chain Reaction (qPCR) Assays

Total RNA was extracted using RNeasy Mini kit (Qiagen, Hilden, Germany). First-strand cDNA was generated from 300 ng of total RNA using Takara PrimeScript RT Master Mix (Takara Bio Inc., Shiga, Japan) following the manufacturer’s recommended protocol. The mRNA expression levels of *GAPDH*, *StAR*, and steroidogenic enzymes such as *CYP11B2*, *CYP17A1*, *HSD3B1*, *HSD3B2*, *CYP21A2* were determined using a Taqman Gene Expression Assay kit (Applied Biosystems, Waltham, MA, USA). mRNA expression levels were analyzed as arbitrary units normalized against *GAPDH* expression.

### 4.3. Cell Culture and Reagents

HAC15 cells were provided by Professor WE Rainey (University of Michigan, Ann Arbor, MI, USA). Cells were cultured in DMEM/F12 with 10% Cosmic Calf serum (HyClone, Logan, UT, USA); detailed methods of cell culture are described in our previous report [59,62]. Ouabain was obtained from Tocris Bioscience (#1076, Ellisville, MO, USA). 

### 4.4. Lentiviral Production and Infection

The lentiviral plasmid pCDH-CMV-MCS-EF1-Puro was purchased from System Bioscience (Palo Alto, CA, USA). The open reading frame of *ATP1A1* gene with L104R mutant was obtained from GenScript (Piscataway, NJ, USA) and ligated into the multiple cloning site of the plasmid. Lentivirus production and infection in HAC15 cells were performed as previously reported [40]. The pCDH-CMV-MCS-EF1-Puro plasmid, which does not have any genes in the multiple cloning site, was used as control.

### 4.5. Transcriptome Analysis

Gene expression analysis was performed using total RNA extracted from adrenal tissues by SurePrint G3 Human Gene Expresson 8 × 60 K v2 (Agilent Technologies Inc,. Santa Clara, CA, USA) as we previously reported [61]. The transcriptome data were analyzed by the R software package (https://www.r-project.org/, accessed on 26 December 2018). Genes expressed in *ATP1A1* mutated APA and NFA were subjected to pathway analysis using Gene Set Enrichment Analysis (GSEA; http://software.broadinstitute.org/gsea/index.jsp, accessed on 10 March 2015). Heatmaps using gene expression by microarray analysis were depicted by the Heatmapper (http://www.heatmapper.ca/, accessed on 10 September 2021). RNA sequencing analysis was performed using total RNA extracted from HAC15 cells as previously reported [63]. 

### 4.6. Immunohistochemical Analysis

Formalin-fixed paraffin-embedded human adrenal tissues were cut into 4 μm sections, processed, and incubated with mouse monoclonal antibodies against human CYP11B2 and rabbit polyclonal antibody against human NKA (#ab58475, Abcam, Cambridge, UK) as previously described [60,63]. Secondary detections for CYP11B2 was performed using SignalStain^®^ Boost IHC Detection Reagent (#8114, Cell Signaling Technology, Danvers, MA, USA). Ultra View Universal DAB Detection Kit^®^ (#760-500, Ventana Medical System Inc., Oro Valley, AZ, USA) was applied for secondary detection for NKA expression. 

### 4.7. Cell Proliferation Assay

HAC15 cells were seeded on 96 well plates. After the cells reached to a confluency of 40–50%, transduction of *ATP1A1* mutation (*n* = 6) or control (*n* = 6) vector was performed using the lentiviral systems described above. The effect of *ATP1A1* mutant on cell proliferation was evaluated by counting cell number using TC20 Cell Counter (Bio-Rad, Hercules, CA, USA) after indicated time. The amount of DNA for cell number estimation was measured by CyQUANT Direct Cell Proliferation Assay Kit (Invitrogen, Carlsbad, CA, USA), which provided an index of cell proliferation, following manufacturer’s protocol [64]. The fluorescence was detected using Varioskan Flash (Thermo Fisher Scientific, Waltham, MA, USA).

### 4.8. Cell Cycle Assay

HAC15 cells were seeded on 6-well plates. After transduction of *ATP1A1* mutant (*n* = 3) or control (*n* = 3), cells were incubated with DMEM-F12 containing 10% cosmic calf serum for 48 h. The cells were treated with 10 μM of cycloheximide for 24 h, because cycloheximide-induced cell cycle arrest in mitotic entry phase. Following the protocol of Cell Cycle Assay Solution Blue (Dojindo Lab., Kumamoto, Japan), the cells (5.0 × 10^5^) were incubated with supplemental solutions. The cells were injected into the flow cytometer instrument (BD FACSAriIIu, BD Biosciences, Franklin Lakes, NJ, USA). 

### 4.9. Metabolite Analysis

After transduction of the *ATP1A1* mutant (*n* = 3) or control (*n* = 3), HAC15 cells were incubated with DMEM-F12 containing 10% cosmic calf serum on 6-well plates for 24 h. Collected cells were washed with 5% mannitol solution and treated with methanol and internal standard solution (Human Metabolome Technologies Inc., Tsuruoka, Japan). Ultrafiltration was performed with a 5-kDa cutoff filter at 9100× *g* and 4 °C for 35 m. Metabolites including PRPP were analyzed by capillary electrophoresis time-of-flight mass spectrometry as reported elsewhere [65]. 

### 4.10. Intracellular Ca^2+^ Detection

Fluo4-AM (Invitrogen, Carlsbad, CA, USA) were applied for the measurements of intracellular Ca^2+^ concentrations as we previously described [59].

### 4.11. Western Blotting

HAC15 cells were seeded on 12-well plates. After transduction of the *ATP1A1* mutant (*n* = 3) or control (*n* = 3) vectors and attainment of confluency, the HAC15 cells were incubated with DMEM/F12 containing 0.1% serum for 24 h. Cells were incubated with fresh media including 10% serum with or without 1 nM of ouabain for 5 m. Cell lysis, sodium dodecyl sulfate-polyacrylamide gel electrophoresis, transfer, and blot processing were performed as reported previously [62]. Immunoblotting analyses using phospho-Src Family (Tyr416) (D49G4) Rabbit mAb (#6943, Cell Signaling Technology, Danvers, MA, USA), Src (36D10) Rabbit mAb (#2109, Cell Signaling Technology, Danvers, MA, USA), and GAPDH (14C10) Rabbit mAb (#2118, Cell Signaling Technology, Danvers, MA, USA) were performed. Phopho-Src levels were analyzed as arbitrary units normalized against Src expression.

### 4.12. Aldosterone Measurement

HAC15 cells were seeded on 24-well plates. After transduction of *ATP1A1* mutant (*n* = 3) or control (*n* = 3) in HAC15 cells and attainment of confluency, cells were serum deprived in DMEM/F12 containing 0.1% serum for 24 h. Cells were incubated with fresh media with 0.1% serum for another 24 h. Aldosterone levels were measured in cell culture supernatants by an ELISA kit as previously described [66]. Cellular protein levels were measured using a Pierce BCA Protein Assay Kit (Thermo Fisher Scientific, Waltham, MA, USA). The aldosterone levels were normalized based on cellular protein levels. Both aldosterone and cellular protein levels were assayed in triplicate.

### 4.13. Statistical Analysis

The results are expressed as mean values with SEM of at least three separate experiments. The statistical significance was analyzed by *t*-test for two groups or by one-way ANOVA followed by Bonferroni comparison for multiple groups. The changes over time between the two groups were tested by two-way ANOVA. Significance level of *p* < 0.05 was considered statistically significant. Statistical analysis was performed using SPSS for Windows (release 24.0; SPSS, Inc, Chicago, IL, USA).

## 5. Conclusions

We demonstrated that APAs with *ATP1A1* mutations exhibited more abundant NKA expression relative to NFA or APAs with *KCNJ5* mutations and that a common *ATP1A1* mutation L104R stimulated cell proliferation in HAC15 cells. Our results also suggest that *ATP1A1* mutation-mediated adrenal cell proliferation is regulated by cardiotonic steroids inducing cell signal transduction involving the increased phosphorylation of Src. Cardiotonic steroids are a potential risk factor for the progression of a ZG cell that develops an *ATP1A1* mutation to an APA and might have pathogenic importance. This study provides a mechanism for APA tumorigenesis and consideration of preventive approaches for *ATP1A1*-mutated APAs.

## Figures and Tables

**Table 1:** Pathway analysis using Gene Set Enrichment Analysis (GSEA) of genes expressed in five aldosterone-producing adenomas (APA) with an *ATP1A1* mutation and five non-functioning adrenocortical adenomas (NFA).

Ranking	Pathway Increased in APA with *ATP1A1* Mutation
1	Cancer Head and Neck vs. Cervical Down
2	Ion Transport by P Type ATPase
3	Proximal Tubule Bicarbonate Reclamation
4	Kidney
5	LG1 Targets Up

The transcriptome data from microarray analysis were analyzed by the R software package. The pathways highly expressed in *ATP1A1* mutated APA in contrast to NFA were detected.

**Table 2:** The difference of genes related with P type ATPase between aldosterone-producing adenoma (APA) with *ATP1A1* mutation and nonfunctioning adrenocortical adenoma (NFA).

Gene Symbol	Fold Increase (vs. NFA)	*p* Value
*ATP2A3*	5.12	0.036
*ATP9A*	4.34	0.004
*ATP2B3*	3.84	0.040
*ATP2B2*	3.71	0.049
*ATP4A*	3.43	0.059
*ATP1B2*	3.17	0.001
*ATP1A2*	2.90	n.s
*ATP8A2*	2.82	0.012
*ATP1A4*	2.13	0.001
*ATP1B1*	2.01	0.001
*ATP1A1*	1.86	n.s
*ATP1B3*	1.67	n.s
*ATP2A1*	1.43	n.s
*ATP2C2*	1.35	n.s
*ATP7B*	1.33	n.s
*FXYD2*	1.32	n.s
*ATP9B*	1.24	n.s
*ATP10D*	1.22	n.s
*ATP10A*	1.10	n.s
*ATP4B*	1.10	n.s
*ATP1A3*	1.10	n.s
*ATP10B*	1.05	n.s
*ATP8A1*	1.03	n.s
*ATP7A*	0.98	n.s
*ATP8B3*	0.93	n.s
*ATP11B*	0.90	n.s
*ATP11C*	0.90	n.s
*ATP12A*	0.87	n.s
*ATP8B1*	0.87	n.s
*ATP2A2*	0.79	n.s
*ATP11A*	0.79	n.s
*ATP2C1*	0.75	n.s
*ATP8B4*	0.41	n.s

The statistical significance was analyzed by *t*-test.

## Data Availability

The data underlying this article will be shared upon appropriate request to the corresponding author.
